# MR Lymphography of Lymphatic Vessels in Lower Extremity with Gynecologic Oncology-Related Lymphedema

**DOI:** 10.1371/journal.pone.0050319

**Published:** 2012-11-28

**Authors:** Qing Lu, Zachary Delproposto, Alice Hu, Christine Tran, Ningfei Liu, Yulai Li, Jianrong Xu, Duy Bui, Jiani Hu

**Affiliations:** 1 Department of Radiology, Renji Hospital, Shanghai Jiao Tong University School of Medicine, Shanghai, China; 2 Department of Radiology, Henry Ford Hospital, Detroit, Michigan, United States of America; 3 Department of Natural Sciences, Michigan State University, East Lansing, Michigan, United States of America; 4 Graduate School of Biomedical Sciences, University of Medicine and Dentistry of New Jersey, Newark, New Jersey, United States of America; 5 Department of Plastic & Reconstructive Surgery, Shanghai 9th People's Hospital, Shanghai Jiao Tong University School of Medicine, Shanghai, China; 6 Department of Radiology, Wayne State University, Detroit, Michigan, United States of America; NIH, United States of America

## Abstract

**Objective:**

To characterize lymphatic vessel morphology in lower extremity lymphedema using MR lymphography at 3T.

**Study Design:**

Forty females with lower extremity lymphedema secondary to gynecologic carcinoma treatment underwent MR lymphography (MRL) at 3T. Lymphatic vessel morphology in normal and affected limbs was compared.

**Results:**

The median diameter of the lymphatic vessels in swollen calf and thigh were significantly larger than that in the contralateral calf and thigh, respectively (p<0.05). The median number of lymphatic vessels visualized in normal calf was less than that in the lymphedematous calf (p<0.01), while no significant difference was found between the normal thigh and swollen thigh. Lymphatic vessel number in the affected calf was significantly greater than that in affected thigh and the mean diameter of affected calf was also significantly wider than that of affected thigh (p<0.01). Mean diameter of lymphatic vessels in the affected calf was significantly different between stage I and stage III (p<0.05), but not significantly different between stages I and II, and between stages II and III (p>0.05). The median number of lymphatic vessels for affected calf showed significant difference between stage I and stage III, and between stage II and stage III (p<0.05), but no significant difference between stage I and stage II (p>0.05). There was no significant difference in mean diameter or median number of lymphatic vessels in the affected thigh found between different stages (p>0.05).

**Conclusion:**

There are significant differences in the number or diameter of lymphatic vessels between normal and affected limbs and there are significant differences for affected calf between early and late stages of lymphedema; therefore, MR lymphography can be helpful in diagnosis or clinical staging for lower extremity with gynecologic oncology-related lymphedema.

## Introduction

Lower extremity lymphedema (LEL) secondary to gynecologic tumor treatment is the result of abnormal accumulation of fluid in the interstitial tissues [1], [2], [3], [4], [5], [6]. Secondary LEL in gynecological cancer survivors is often caused by injury to the lymphatic draining systems occurring during surgery [1], [4], [5], [6]. It has also been demonstrated that iatrogenic secondary LEL can occur after stripping of veins, liposuction, arterial revascularization procedures, or even from the biopsy of a single lymph node [7], [8], [9], [10]. To prevent or minimize the occurrence of lymphedema, the lymphatic drainage system in the region of interest for each individual patient must be mapped. Detailed examination of the lymphatic drainage system may also provide useful information for treatment evaluation of patients with secondary LEL [11].

Magnetic resonance lymphangiography (MRL) is a relatively new MR technique that can assess lymphatic networks through subcutaneous injection of commonly used contrast agents [12], [13], [14], [15]. However, the majority of MRL research has been confined to primary lymphedema which may have different MRL characteristics from secondary LEL because of etiological differences [16]. There have no comprehensive prior studies evaluating MRL in the lower extremity. The few existing studies with MRL do not evaluate the morphology of lymphatic vessels in normal lower extremities, or study the relationship between abnormal lymphatic vessels and the severity of lymphedema [17], [18], [19]. Therefore, the purpose of this study is to characterize normal and abnormal lymphatic vessels in patients with secondary lymphedema using high spatial resolution MRL at 3T.

## Materials and Methods

### Ethics Statement

All research procedures were approved by the Institutional Review Board of the Shanghai Jiaotong University School of Medicine and were conducted in accordance with the Declaration of Helsinki. Written informed consent was obtained for all patients.

### Study design

From June 2008 to March 2011, forty (40) female patients from a pool of 46 patients with clinically diagnosed lower limb edema secondary to gynecologic carcinoma treatment were enrolled in this study. Six (6) patients were excluded from the evaluation due to recurrent tumor (2 patients), deep vein thrombosis (3 patients), or lipolphymphedema (1 patient). The mean age was 52.3±11.9 years (range: 27–74 years). The mean duration of limb swelling in this cohort was 1.8±1.2 years, (range: 1 month to 7 years). The lymphedema characteristics, along with gynecologic carcinoma type and treatment modality, are summarized in **Table 1**. Clinical diagnosis and staging was performed by an experienced lymphologist (NFL) with over 20 years of experience. Clinical staging was based on the criteria set forth in the consensus document of the International Society of Lymphology 2009 [20]. Of the forty patients, thirty-two had unilateral lower limb extremity lymphedema and eight had bilateral lower limb lymphedema. Of the 48 swollen limbs, 10 were in stage I, 22 in stage II, and 16 in stage III. Exclusion criteria were patients with contraindications for an MRI, leg edema secondary to a recurrent tumor, deep vein thrombosis, chronic vascular disease, renal disease, lipolymphedema and any other medical comorbidity induced LEL other than gynecologic cancer treatment.

**Table 1:** Carcinoma diagnosis and treatment, and lymphedema characteristics in 40 patients.

Clinical Characteristic	No. of patients (%)
**Type of gynecological malignancy**	
Cervical cancer	24 (60%)
Endometrial carcinoma	13 (32.5%)
Ovarian cancer	3 (7.5%)
**Therapeutic modalities**	
Abdominal radical hysterectomy	33 (82.5%)
Laparoscopic radical hysterectomy	7 (17.5%)
**Adjuvant therapy**	
None	7 (17.5%)
External beam radiotherapy	23 (57.5%)
Chemotherapy	5 (12.5%)
Radiotherapy adds Chemotherapy	5 (12.5%)
**Symptomatic lymphedema onset after treatment**	
≤6 months	5 (12.5%)
>6months – 3 years	22 (55%)
>3 years	13 (32.5%)

### Contrast agent and material administration

Gadopentate dimeglumine (Gd-DPTA) (Magnevist, Bayer Schering Pharma AG, Berlin, German) is a water-soluble, small molecular weight (1 kDa), paramagnetic contrast agent with a gadolinium (Gd) concentration of 0.5 mol/L. This contrast agent is not subject to metabolization, is excreted unchanged by passive glomerular filtration, and causes very minor tissue damage after non-intravenous injection or extravasation [12], [15]. Therefore, Gd-DPTA offers an acceptable safety profile for intracutaneous administration. A 24 gauge needle was used for injection in this study. A total of 5.5 mL of contrast material and 0.5 mL mepivacainhydrochloride 1% were subdivided into six portions and injected intracutaneously into the dorsal aspect of each foot in the region of the three interdigital webs [12]. The volume injected into each point was about 1 ml. Mepivacainhydrochloride 1% was administered with the contrast agent to alleviate the pain at the injection site. After contrast material injection, a 30 second massage was performed.

### MR imaging

All MR examinations were performed on a clinical 3.0 T MR imaging device (Achieva, Philips Medical Systems, Best, Netherlands) with a six-element phased-array coil. Each patient was placed in the supine position with feet first. Imaging started beginning with the foot and progressed towards the inguinal region in three separate and successive acquisitions.

For interstitial MRL, 3D T1 high-resolution isotropic volume excitation with fat saturation (THRIVE) was acquired before the intracutaneous administration of Gd-DTPA contrast and 20 minutes after the administration. The MR imaging parameters were as follows: TR/TE: 3.5/1.7, flip angle: 25, FOV: 360 cm×320 cm, matrix: 300×256, slices: 55–95, voxel size: 1.4 mm×0.5 mm×0.5 mm, acquisition time: 3 min. Previous studies have indicated that 20 minutes is the optimal time for observation of lymphatic systems after Gd administration [13]. THRIVE acquisitions were performed sequentially at the level of the calves, knees, and inguinal region. To outline the vessels, the 3D MRL images were then reconstructed from the post-contrast coronal images using a Maximum Intensity Projection (MIP) technique. Overall examination time was approximately thirty minutes.

### Image interpretation and data analysis

Two radiologists (QL with 15 years of experience and JRX with over 20 years of experience) examined all images blind to lymphedema staging. In the event of disagreement, agreement was reached by consensus. The appearance, distribution pattern, and morphologic characteristics of the lymphatic networks, including number of dilated vessels, maximum transversal diameter and dermal backflow (irregular, patchy shaped, high signal intensity area with blurred borders on MRL imaging) were analyzed in both extremities. The number of lymphatic vessels and maximum transversal diameter of lymphatic vessels at the region of the calf and thigh were compared between the affected limb and the contralateral normal limb for 40 patients. Lymphatic vessels were identified based on their twisted and beaded-appearance. Therefore, lymphatic vessels may be easily distinguished from vascular vessels, which render a linear appearance with low signal intensity [14], [21]. All results were expressed as medians and means with corresponding standard deviations. The Student's T test and Wilcoxon rank tests were applied to determine the significance of differences between mean values for the maximum transversal diameter and the median numbers of lymphatic vessels, respectively. Further comparisons were made between stages I, II and III using the above metrics. ANOVA was used to determine whether the differences between the three stages were significant. A value of p<0.05 was determined to indicate a significant difference.

## Results

**Figure 1:**
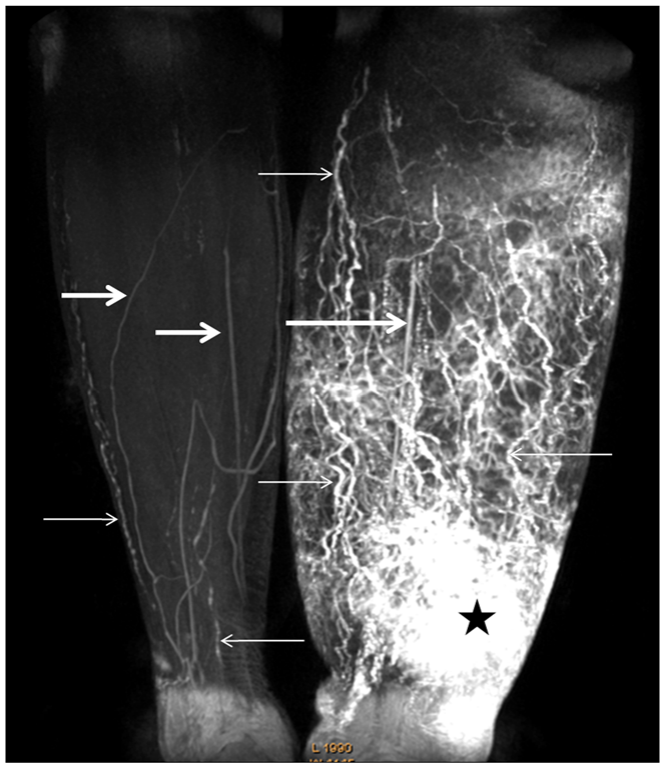
Contrast-enhanced 3D MRL image of calves. Contrast-enhanced 3D frontal MR Lymphography, Maximum intensity projection image obtained 20 min after contrast injection of a 57-year-old woman with stage III left LEL and who underwent radical hysterectomy following adjuvant radiotherapy. Image shows numerous dilated lymph vessels with a beaded appearance in the left calf (small arrows). In the right normal calf, unaffected lymphatic vessels show an ill-defined intermittent outline (small arrows). The patterns of dermal back-flow showed an irregular patchy shaped high intensity area with blurred borders identified above the left ankle (star). The concomitantly enhanced vein shows a linear structure with lower signal intensity (large arrows).

Lymphatic vessels in the 32 normal lower extremities showed an intermittent, low signal intensity line on MRL and rendered ill-defined outlines on MIPs (**Figure 1** **,** **2**). In contrast, lymphatic vessels in the 48 affected lower extremities were visualized as beaded, dilated, high signal intensity vessels on MRL with sharply defined outlines on MIPs (**Figure 1** **,** **2**). The mead transverse diameter of the lymphatic vessels for the normal and swollen calf was 2.49±0.79 mm and 3.41±1.4 mm (t = 3.364, p = 0.003), and 1.29±0.35 mm and 2.11±1.25 mm (t = 3.60, p = 0.002) for the normal and swollen thigh, respectively. The median number of lymphatic vessels visualized in the normal and lymphedematous calf was 7 and 10 (z = −4.026, p<0.01), while for the thigh it was 5 and 5 (z = −0.198, p = 0.843), respectively. The number of lymphatic vessels in affected calf was significantly greater than that in the affected thigh (z = −4.970, p<0.01) using Wilcoxon statistical method. The mean diameter of the affected calf was 3.41±1.4 mm, which was also significantly wider than that of the affected thigh, 2.11±1.25 mm (t = 4.398, p<0.01).

**Figure 2:**
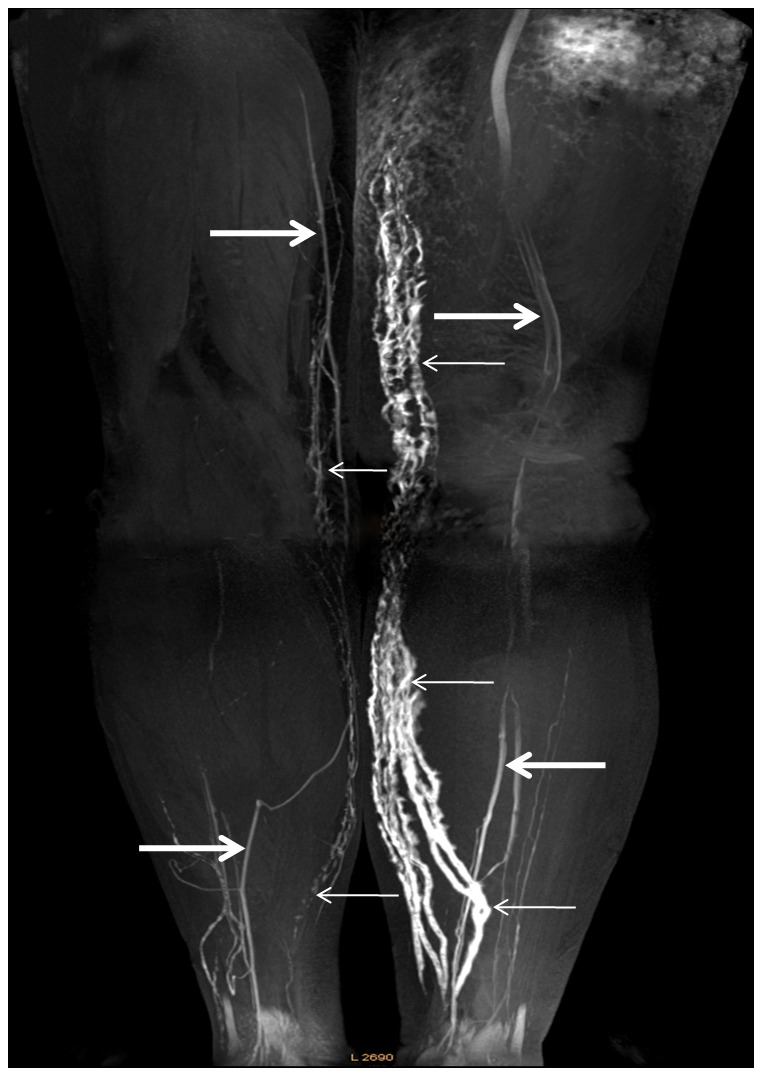
Contrast-enhanced 3D MRL image of the lower extremities. Coronal 3D MR Lymphography image of the lower extremities obtained from 20 min to 23 min after subcutaneous injection of contrast material into a 74-year-old female who underwent abdominal hysterectomy for the treatment of endometrial carcinoma. Her left extremity became swollen one year following surgery. Several abnormal dilated lymph vessels are clearly delineated in the left calf, which extend to the inner thigh (small arrows). In the contralateral normal extremity, a few lymphatic vessels show a discontinuous outline with relatively low signal intensity (small arrows). The concomitantly enhanced vein shows a linear structure with lower signal intensity (large arrows).

The mean diameter and median number of lymphatic vessels visualized for affected calf and thigh at different clinical stages are summarized in **Table 2**. For the affected calves, the mean diameters of lymphatic vessels for each clinical stages showed significant differences using ANOVA (F = 3.898, p = 0.027) Further intra-stage comparison produced p-values of 0.365 for stages between I and II, 0.367 for stages between II and III, and 0.024 for stages between I and III. No significant difference was found for inter-stage comparisons of mean transversal lymphatic vessel diameter visualized for the affected thigh (F = 2.093, P = 0.135). ANOVA variance analysis was applied to make comparisons between the median numbers of lymphatic vessels visualized in various clinical stages for the affected calf, which showed a significant difference (F = 5.115, P = 0.010). Further intra-stage comparison produced p-values of 1.0 between stages I and II, 0.037 between stages II and III, and 0.019 between stages I and III. No difference was found for inter-stage comparisons of number of vessels visualized for the affected thigh (F = 0.078, P = 0.925).Dermal back flow was identified in 25 (52.1%) of the 48 affected extremities (**Figure 2**). The patterns of dermal back-flow appeared as an irregular, patchy shaped, high signal intensity area on MRL.

**Table 2:** The number and diameter of lymphatic vessels in patient with different stages.

Stages	N	Diameter (mean ± SD) mm		Number (Median)	
		Calf	Thigh	Calf	Thigh
**Stage I**	10	2.55±1.01	1.83±0.81	9.5	4
**Stage II**	22	3.35±1.22	1.94±1.2	12	4.5
**Stage III**	16	4.04±1.60	2.73±1.58	19	5

## Discussion

Using high spatial resolution MRL at 3T, we were able to visualize lymph vessels of the lower extremity in patients with secondary lymphedema due to gynecologic tumor treatmentand evaluate their morphologic characteristics. To our knowledge, no systematic comparison has been performed on the diameter or number of lymph vessels between calf and thigh, or between different stages of secondary LEL. Our study indicates that morphologic changes due to secondary LEL are more prominent in affected calf than the affected thigh. There were significant differences in the number of lymphatic vessels identified in the affected calf between stages I and III (p = .019) and stages II and III (p = 0.037). There were significant differences in the diameter of lymphatic vessels in the affected calf between stages I and III (p = 0.024). However, no differences were found between the inter-stage comparisons of the above metrics for the affected thighs. Moreover, there was a significant difference in both transverse diameter and number of lymphatic vessels between normal and affected calf, but no significant difference between normal and affected thighs. This observation suggests that early and accurate diagnosis of secondary LEL should focus on the calf rather than the thigh. It also implies that lymphatic neoperfusion or neovascularization occurs more frequently in the affected calf than the affected thigh. In contrast, patients with congenital or praecox types of primary lymphedema are reported to have hypoplastic lymphatic vessels in the lower extremities [16]. Our findings are consistent with the classic scenario of secondary LEL development. For patients with early stage disease, the most common initial symptom is a painless, soft and reversible pitting edema on the distal part of the extremity. With disease progression, the edema spreads proximally and lymphatic vessels in the thigh become involved [16], [22]. Our results demonstrated that the number and diameter of lymphatic vessels in affected calf consistently correlate with disease severity, although this is not true for the thigh. It is worth noting that the calf result is consistent with a previous study on secondary LEL using lymphoscintigraphy [23]. However, our result disagrees with with a previous study of primary lymphedema, where it was found that the degree of lymphatic vessel dilatation did not correlate with severity of lymph drainage dysfunction [13]. This difference in observation may reflect underlying differences in pathogenesis between primary and secondary lymphedema.

Although a few of our findings contradict reported characteristics of primary lymphedema, most of our findings show the parallels between primary and secondary lymphedema. Abnormal lymphatic vessels showed a beaded appearance in affected extremities for all 40 patients, which is in agreement with previous studies using MRL to study primary lymphedema [13], [14], [21]. Dermal back-flow or abnormal distribution of contrast agent in subcutaneous tissues was also identified in 52% of affected extremities, although its prevalence is similar in primary lymphedema, which ranges between 25% to 75% [14], [17], [21], [24]. The presence of dermal back-flow suggests obstruction of proximal lymphatic flow. It has been proposed that the obstruction is likely due to acquired damage or destruction of the lymphatic network for secondary LEL. In primary lymphedema, however, it is likely due to hypoplastic vessels or a deficient valvular system [25].

The importance of preventing lymphatic injuries in surgery has long been recognized in terms of both early complications, such as lymphorrhea, lymphocele, wound dehiscence, or infections, and late complications such as lymphangitis or lymphedema [26]. For example, the potential of acquiring secondary lymphedema in the extremity after node dissection is quite high [3]. The incidence of lymphedema in the operated leg is 43% after radical groin dissection and 37% after superficial groin dissection [27]. Thus, mapping the lymphatic drainage system of each individual patient could potentially prevent or minimize secondary LEL due to surgical procedures. Our results have clearly demonstrated the capability of MRL to visualize lymph vessels of normal and affected lower extremity. It is also worth noting that high spatial resolution is crucial for visualizing subtle lymphatic vessels in healthy extremities [3], [28] because the diameters of lymphatic vessels are significantly smaller than those in affected edematous extremities. High-field strength MR systems (e.g., 3.0 T) are preferably over lower magnetic strengths due to improved signal-to-noise ratios. Lymphoscintigraphy, the most commonly used technique for imaging lymphatic system in clinical practice today, cannot reveal the subtle structure of lymph vessels particularly in normal lower extremity [29], [30]. High quality images of the lymphatic drainage system could also be useful for patients already diagnosed with secondary lymphedema [11], [31], [32]. Prophylactic anastomosis is a surgical procedure used to prevent the development of lymphedema in the normal limbs of patients with unilateral lymphedema [11]. Lymphaticovenous anastomosis, on the other hand, is a surgical procedure to treat secondary lymphedema, especially in earlier stages of disease [32]. The exclusion for both procedures is patients with distal or obliterative disease, and those with combined pelvic and distal obliterative disease. If the recently developed supermicrosurgical technique is used, the caliber size of lymphatic vessels selected for precise anastomosis must be more than of 0.3 mm [33]. In addition, outcomes are poor for patients with atrophied lymphatic vessels [11]. Therefore, anatomic delineation of the lymphatic drainage system in the targeted limb, including the number, caliber, location, and distribution of lymphatic vessels should be carefully evaluated before surgery because affected lymphatic vessels are often dilated and sclerotic, they weakly visible or even not visible with staining during microsurgery. These could be addressed with high spatial resolution MRL, which has the capability to precisely map lymphatic drainage routes and thereby help aid patient selection and surgical planning.

MRL has the potential to become a useful tool in planning and predicting the success of lymphatic microsurgery. Multiple lymphatic-venous anastomoses (LVA) is an accepted approach for the microsurgical treatment of peripheral lymphedema based on clinical experience and multiple research studies [34]–[36]. Lymphoscintigraphy has an essential role in microsurgery both for pre-surgical evaluation and for post-treatment monitoring [34], [35]. Previous research has shown that MRL was more sensitive and accurate than lymphoscintigraphy in the detection of anatomical abnormalities in the lymphatic system in patients with extremity lymphedema [21]. Therefore, we propose that detailed anatomic delineation of the lymphatic drainage system in the targeted limb provided by MRL, as performed in this study, should be useful for surgical planning. MRL has also been proven to be a safe and accurate imaging method for postoperative evaluation of the lymphatic circulation in patients undergoing microlymphatic surgery with essentially no risk, due to the minimal invasiveness of the procedure and lack of ionizing radiation [31]. Prior to multiple LVA surgery, Doppler ultrasound is typically used to identify possible venous disorders associated with lymphedema because in most patients, venous dysfunction must be corrected during surgery (e.g., performing valvuloplasty in cases of venous insufficiency). In other cases, venous vascular evaluation contraindicated derivative lympho-venous shunts but at the same time facilitated referral of the patient for reconstructive microsurgical operations [34]. Due to the use of an extracellular, water-soluble, low-molecular weight contrast agent, communicating veins enhanced and were clearly visualized on MRL in all subjects. Although small venous and lymphatic structures can have similar, and confusing, characteristics, detailed structural information and the spatial relationship with lymphatic vessels can be readily discerned. Therefore, a single MRL examination may mitigate the need for the combination of both lymphoscintigraphy and Doppler ultrasound evaluations.

There are several limitations to our study. Due to the use of an extracellular, water-soluble, low-molecular weight contrast agent, a substantial amount of contrast material was eliminated via the circulatory system. The volume of contrast material remained in lymphatic system was insufficient to enhance the iliac lymphatic vessels in most patients; which is a limitation given that pelvic lymphatic vessels are in the most likely area where causative obstruction might be. Presently, though, microsurgical multiple lymphatic-venous anastomoses are performed in the subinguinal region in pateints with lower extremity lymphedema [34], [36]. We believe that newer paramagnetic macromolecular contrast agents with high lymph affinity may overcome this problem in the future. Furthermore, serial MRL examinations were not performed during the course of lymphedema treatment and therefore we are unable to evaluate the sensitivity and specificity of MRL. Lastly, there were only a small number of cases for each clinical stage.

In conclusion, high spatial resolution MRL provides anatomical and morphological information of the peripheral lymphatic system and permits accurate characterization of lymphatic drainage system, which may help diagnosis, surgical planning and treatment evaluation of LEL patients after gynecologic oncology-related treatment.
